# Chemical Profile and Evaluation of the Growth-Inhibitory, Anti-Inflammatory, and Antioxidant Activity Potential of Polar Extracts of *Reseda alba* L. (Resedaceae)

**DOI:** 10.3390/plants15121821

**Published:** 2026-06-12

**Authors:** Giuseppe A. Malfa, Antonietta Cerulli, Donata Condorelli, Assunta Napolitano, Elena Preite, Nicodemo G. Passalacqua, Monica R. Loizzo, Sonia Piacente, Rosa Tundis

**Affiliations:** 1 Department of Drug and Health Sciences, University of Catania, Viale A. Doria 6, 95125 Catania, Italy; gmalfa@unict.it (G.A.M.); dcondorelli01@gmail.com (D.C.); 2 Department of Pharmacy, University of Salerno, 84084 Fisciano, Italy; acerulli@unisa.it (A.C.); anapoli@unisa.it (A.N.); piacente@unisa.it (S.P.); 3 Department of Pharmacy, Health and Nutritional Sciences, University of Calabria, 87036 Rende, Italy; elena.preite@unical.it (E.P.); monica_rosa.loizzo@unical.it (M.R.L.); 4 Department of Biology, Ecology, and Earth Science, University of Calabria, 87036 Rende, Italy; nicodemo.passalacqua@unical.it

**Keywords:** *Reseda alba*, leaves, flowers, stems, immature fruits, LC-ESI/HRMS/MS, cancer cells, LDH

## Abstract

This study provides a comprehensive evaluation of the chemical composition and the biological properties of *Reseda alba* L., commonly known as white mignonette. Extracts obtained from leaves (L), flowers (F), stems (S), and immature fruits (Fr) by ultrasound-assisted extraction (UAE) were assessed for their antioxidant, anti-inflammatory, and growth-inhibitory activity, and chemically characterized by an analytical approach based on liquid chromatography/electrospray/high-resolution tandem–mass spectrometry (LC-ESI/HRMS/MS). The resulting chromatographic profile revealed 30 major constituents belonging to the flavonoids, glucosinolates, phenolic acids, and polar lipids, as well as hydroxy fatty acid classes. Naringenin-di-*C*-glucoside, isorhamnetin-*O*-deoxyhexosyl-hexoside, kaempferol-*O*-dideoxyhexosyl-hexoside, and isorhamnetin *O*-dideoxyhexoside are reported here for the first time in the genus *Reseda*. The Fr extract exhibited the highest anti-inflammatory and radical scavenging properties, likely due to its higher flavonoid content compared to the other extracts. On the other hand, the F extract significantly reduced the viability of colorectal adenocarcinoma (CaCo-2) and hepatocarcinoma (HepG-2) cells. Lactate dehydrogenase (LDH) release assay showed that the treatments with *R. alba* did not induce the release of the marker enzyme in CaCo-2 and HepG-2 cells, suggesting the involvement of a different cell death pathway. Overall, the bioactivities observed among the different plant organs highlight the beneficial potential of *R. alba* and provide a rationale for future bioactivity-guided isolation studies.

## 1. Introduction

Cancer is a major global health problem and one of the leading causes of death worldwide. It includes over 200 types that require extensive healthcare resources for diagnosis and treatment [1]. Among them, colorectal cancer is still a major global burden, ranking third in incidence and second in cancer mortality, with 1.9 million new cases reported in 2020 [2]. Its distribution varies geographically and is influenced by daily habits, with increasing rates among younger individuals in developed countries [3]. Liver cancer, primarily hepatocellular carcinoma, is also a leading cause of cancer death, with over 900,000 new cases annually. It is most prevalent in territories with high exposure to viral hepatitis and other risk factors, while its rise in Western countries is linked to metabolic disorders.

Despite significant progress in treatment, prognosis remains poor due to late diagnosis and frequent recurrence. One of the most common malignancies in women is breast cancer, mainly treated with surgery and systemic therapies. However, the efficacy of chemotherapy is often limited by drug resistance, side effects, and recurrence [1], prompting growing interest in natural compounds for their multi-target activity and lower toxicity.

Notwithstanding the recent advances in the field of chemically synthetic/synthesized compounds, nature remains the main supplier of bioactive molecules.

The research of natural products is a valuable approach for the discovery and development of novel biologically active compounds possessing unique structures and mechanisms of action [4]. Natural compounds have been shown to mediate, modulate, or enhance the action of anti-cancer drugs by affecting DNA damage repair mechanisms, cell cycle progression, apoptosis, migratory properties, the immune response, and other processes. Moreover, they act as sensitizers of anti-cancer treatments by reducing therapy resistance and/or lowering adverse effects. The antioxidant, anti-inflammatory, and immunomodulatory effects of natural compounds can further support anti-cancer treatments by reducing side effects.

*Reseda* is the largest genus of the family Resedaceae [5]. Many *Reseda* species are restricted to the Mediterranean basin, while four species, such as *R. alba*, *R. lutea*, *R. luteola*, and *R. phyteuma* are distributed worldwide [6]. Several *Reseda* species have been investigated for their bioactivity, revealing promising anti-inflammatory, antioxidant, cytotoxic, and antibacterial properties [5,7,8,9,10,11,12]. Among these, *R. lutea*, also known as *R. vulgaris*, is the most investigated species. It is known not only for its antioxidant and antidiabetic activities but also for its potential anti-cancer properties [13,14,15].

In the present work, the aerial parts (leaves, flowers, stems, and immature fruits) of *R. alba* have been investigated for their chemical profile and bioactivity. *R. alba* is an annual or perennial species, known as white mignonette or white upright mignonette. The species’ native distribution includes numerous countries and territories bordering the Mediterranean Sea and adjacent regions, from Morocco and Spain to Lebanon and Syria. The leaves are deeply divided into many lanceolate–oblong lobes. The inflorescence is a dense, terminal, spike-like raceme that can occupy a significant portion of the upper stem. The fruit is an erect capsule, typically cylindric to ovoid–oblong in shape. Recently, this *Reseda* species has been investigated for its phytochemical composition and its potential inhibitory effects on enzymes (tyrosinases, cholinesterases, and α-amylase), antioxidant activity, as well as diuretic activity [16,17].

To the best of our knowledge, no previous studies have explored the cytotoxic and anti-inflammatory potential activities of *R. alba*, making the present work the first report on these biological effects. Accordingly, this study was designed to evaluate, for the first time, the growth-inhibitory effects of *R. alba* leaf, flower, stem, and immature fruit extracts on the human breast cancer cell line MCF-7, human colon carcinoma cells CaCo-2, and human hepatoma cell line HepG-2. The in vitro antioxidant and anti-inflammatory effects by applying a multi-target approach were also studied. In parallel, a comprehensive and comparative liquid chromatography/electrospray/high-resolution tandem–mass spectrometry (LC-ESI/HRMS/MS) characterization of the polar extracts of *R. alba* was performed to assess *R. alba* extracts as a potential source of bioactive compounds with health-promoting applications, particularly in the context of diseases with high social impact, such as cancer.

## 2. Results and Discussion

### 2.1. Extraction Yield and Chemical Profile

Flowers (F), leaves (L), stems (S), and immature fruits (Fr) of *R. alba* were subjected to ultrasound-assisted extraction (UAE) using methanol as solvent.

UAE is a novel method, belonging to the so-called green chemistry that enables the extraction of bioactive molecules requiring lower energy costs and amounts of solvent, and preserving the integrity of these compounds [18]. In recent years, UAE has been used for the extraction of different bioactive compounds such as phenolic compounds, terpenes, and polysaccharides, among others [19]. Herein, the best extraction yields were obtained with flowers and leaves with values of 12.2 and 11.7%, respectively (Table 1).

The extracts of *R. alba* were analyzed for their total content of phenols, flavonoids, and carotenoids (Table 1). Spectrophotometric determination of the total phenol and flavonoid content evidenced the following trend: immature fruits (Fr) > stems (S) > leaves (L) > flowers (F). The highest amount of both phenolic and flavonoid compounds was detected in the fruit extract (Fr) with a TPC of 30.9 mg chlorogenic acid equivalents/g of plant materials and a TFC of 17.7 mg quercetin equivalents/g of plant materials. Differently, the lowest values were found in F (flower extract) with a TPC of 10.2 mg chlorogenic acid equivalents/g of plant materials and a TFC of 7.8 mg quercetin equivalents/g of plant materials. The TCC is in the range 2.6–6.1 mg equivalents of β-carotene/g of plant materials.

**Figure 1:**
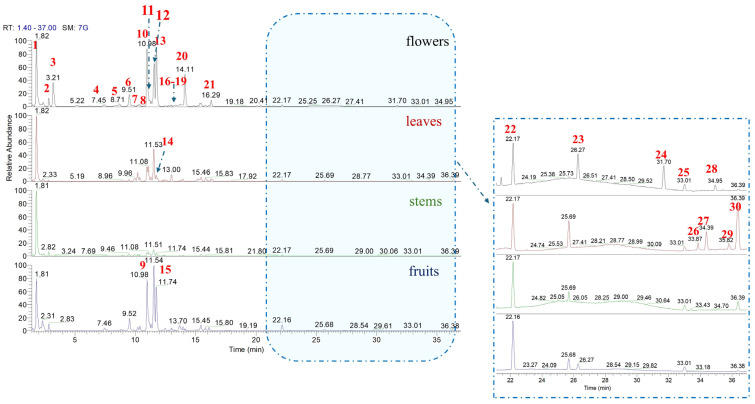
LC-ESI/HRMS profile of *R. alba* extracts in negative ion mode.

To characterize the constituents of *R. alba* extracts, an analytical approach based on liquid chromatography/electrospray/high-resolution tandem–mass spectrometry (LC-ESI/HRMS/MS) was applied. Analyses were performed in negative ionization mode, enabling the detection of analytes either as deprotonated molecules ([M − H]^−^) or as formic acid adducts ([(M + FA) − H]^−^). The resulting chromatographic profile (Figure 1) revealed the presence of 30 major constituents (Figure 1 and Table 2).

Putative identification of metabolites was performed based on LC-ESI/HRMS/MS data, fragmentation patterns, and comparison with reports from the literature.

A summary of the identified compounds—including retention times, molecular ion *m*/*z* values (negative mode), and characteristic MS/MS fragments—is provided in Table 2.

All compounds were detected within the first 37 min of the chromatographic run and were mainly identified as flavonoids, glucosinolates, phenolic acids, polar lipids, and hydroxy fatty acids. The chromatographic method enabled the detection of sucrose (**1**), with a *m*/*z* value of 341.1079 [20]. The predominant class of compounds detected by LC-ESI/HRMS/MS consisted of glycosylated flavonoid derivatives (compounds **5**, **8**–**15**, **17**, and **20**), as supported by their characteristic MS/MS fragmentation patterns [21]. In particular, the identification of compounds **8**–**15**, **17**, and **20** was supported by their retention times and characteristic MS/MS fragment ions. Diagnostic ions were observed at *m*/*z* 301 (compound **8**, corresponding to the quercetin aglycone), *m*/*z* 285 (compounds **9**, **11**–**15**, and **20**, corresponding to the kaempferol aglycone), and *m*/*z* 315 (compounds **10** and **17**, corresponding to the isorhamnetin aglycone), consistent with the respective flavonoid aglycones [22,23,24] (Table 2). The identification of these compounds as *O*-glycosylated flavonoids was supported by the occurrence of characteristic product ions arising from neutral losses of 162 Da (hexose), 146 Da (deoxyhexose), and 132 Da (pentose), together with a neutral loss of 308 D indicative of the concomitant loss of hexose and deoxyhexose moieties [22] (Figure S1). Differently, the characteristic product ions detected at *m*/*z* 475.1239 [M − H − 120]^−^, 457.1118 [M − H − 120 − 18]^−^, 415.1026 [M − H − 120 − 60]^−^, 385.0922 [M − H − 210]^−^, and 355.0712 [M − H − 240]^−^ in the tandem mass spectrum of compound **5**, along with the absence of the aglycone main peak, suggested for this metabolite a di-*C*-glycoside nature [23,24]. Among the flavonoid derivatives, compounds **8**, **13**, **15**, and **20** were previously reported in *R. alba* flowers [16,25,26], while compounds **12** and **14** were identified in *R. luteola* aerial parts [15,27,28].

Compounds **5**, **10**, **11**, and **17** are reported here for the first time in the genus *Reseda*.

Notably, LC-ESI/HRMS/MS analysis revealed the presence of glucosinolate derivatives (compounds **3**, **4**, **6**, and **16**) (Table 2). Glucosinolates are sulfur- and nitrogen-containing secondary metabolites widely distributed in plants. Structurally, they share a common β-D-thioglucopyranose moiety (C_6_H_11_O_5_S), while structural diversity arises from variations in their aglycone side chains. Based on the nature of these side chains, glucosinolates are generally classified as alkyl, aliphatic, alkenyl, hydroxyalkenyl, aromatic, or indole derivatives [29]. The above-mentioned compounds exhibited diagnostic fragment ions at *m*/*z* 75, 97, and 259, corresponding to [C_2_H_3_SO]^−^, [HSO_4_]^−^, and [C_6_H_11_O_9_S]^−^, respectively [30]. Accordingly, compounds **3**, **4**, **6**, and **16** were putatively assigned as glucoconringiin, butyl-glucosinolate, glucotropaeolin, and gluconasturtiin, respectively. The reported glucosinolate compounds were previously described in *R. alba* extracts [5,25].

Moreover, putative annotation of phenolic glycoside derivatives was performed based on LC-ESI/HRMS/MS data and comparison with reports from the literature.

Compound **7** was tentatively assigned, based on MS/MS fragmentation patterns, as vanillic acid-*O*-hexoside [20], and is reported here for the first time in a *Reseda* species. In addition, the phenylpropanoid derivative coniferyl alcohol-*O*-deoxyhexoside (**18**) is reported here for the first time in this genus [31]. Furthermore, LC-ESI/HRMS/MS profiling revealed the presence of azelaic acid (**19**), nonanedioic acid, and 6-gingerol (**22**) identified through MS/MS analysis and reported here for the first time in the genus *Reseda* [20,32].

Finally, LC-ESI/HRMS/MS analysis enabled the tentative assignment of primary metabolites in the extracts. These compounds included a lyso-phosphatidylcholine (**23**) and several oxylipins (**21**, **24**–**30**). 

The LC-ESI/HRMS/MS spectra acquired for these latter compounds displayed characteristic fragmentation patterns of oxylipins. This class of hydroxylated fatty acids differs in the degree of unsaturation and number of hydroxyl groups, and is derived from the oxidative metabolism of essential polyunsaturated fatty acids (PUFAs), such as α-linolenic acid (ALA, 18:3 ω-3) and linoleic acid (LA, 18:2 ω-6).

According to data from the literature, the positions of hydroxyl groups and double bonds along the fatty acyl chain can be tentatively assigned based on characteristic product ions and diagnostic neutral losses generated by molecular rearrangements involving both the head and terminal portions of the acyl chain. Accordingly, compound **21** was tentatively identified as 9,12,13-trihydroxyoctadecenoic acid, for the first time reported in the genus *Reseda*. Regioisomeric hydroxy fatty acids (HFAs) have the same molecular mass and generate identical deprotonated ions ([M − H]^−^) under negative electrospray ionization. Therefore, they cannot be distinguished at the MS^1^ level. However, tandem mass spectrometry (MS/MS) reveals distinct and reproducible fragmentation patterns that enable identification of the hydroxyl position. In particular, a position-specific α-cleavage generally occurs adjacent to the carbon bearing the hydroxyl group.

According to the literature, the simultaneous observation of an intense neutral loss of water (−18 Da) and a prominent fragment at *m*/*z* 59 or 71 provides strong evidence for β-hydroxylation (3-hydroxy fatty acids). In contrast, detection of a fragment at *m*/*z* 75, together with weak or limited dehydration, supports α-hydroxylation (2-hydroxy fatty acids) [33,34]. Based on these fragmentation patterns, compounds **24**–**30** were structurally assigned as reported in Table 2. This is the first report of this class of compounds [34] in the genus *Reseda*. Finally, analysis of compound **23** indicated that it belongs to the lysophosphatidylcholine (l-PC) class, based on the presence of a diagnostic ion at *m*/*z* 184, corresponding to a phosphocholine unit [24]. In negative ion mode, the compound also showed a major fragment corresponding to a neutral loss of 60 Da (C_2_H_4_O_2_), which can be attributed to the formation of the [(M − 15) − H]^−^ ion via methyl loss from the choline head group and concomitant release of neutral methyl formate, consistent with the reported formation of [(M + FA) − H]^−^ formate adducts for phosphatidylcholine derivatives [24]. To the best of our knowledge, this is the first report of an LPC in the *Reseda* genus.

**Table 2:** Metabolites identified in *R. alba* extracts by LC-ESI/HRMS/MS analysis.

n.	R*_t_*	Mol Formula	[M − H]^−^	Δppm	MS/MS	Name	F	L	S	Fr	Reference
**1**	1.82	C_12_H_22_O_11_	341.1079	0.12	179.0549	Sucrose	x	x	x	x	[20]
**2**	2.34	C_9_H_19_O_11_P	333.0586	−3.58	241.0111, 152.9946, 78.9577	Glycerophosphoryl inositol	x	x	x	x	[35]
**3**	3.21	C_11_H_21_O_10_NS_2_	390.0525	0.45	259.0125, 195.0322, 96.9587, 74.9896	Glucoconringiin	x	x	-	x	[30]
**4**	7.45	C_11_H_21_O_9_NS_2_	374.0579	1.31	274.9897, 259.0126, 195.0327, 96.9587, 74.9896	Butyl-glucosinolate	x	x	x	x	[30]
**5**	8.97 *	C_27_H_32_O_15_	595.1666	1.37	475.1239, 457.1118, 415.1026, 385.0922, 313.0712	Naringenin-di-*C*-glucoside	x	x	x	x	[23]
**6**	9.51	C_14_H_19_O_9_NS_2_	408.0418	0.59	259.0128, 96.9587, 74.9896	Glucotropaeolin	x	x	-	-	[30]
**7**	9.91	C_14_H_18_O_9_	329.0873	1.74	167.0337	Vanillic acid-*O*-hexoside	x	x	x	-	[20]
**8**	10.20	C_27_H_30_O_16_	609.1452	0.31	301.0493	Rutin	x	x	x	x	[29]
**9**	10.98	C_27_H_30_O_15_	593.1495	−1.04	285.0542	Kaempferol-*O*-rutinoside	x	x	x	x	[24]
**10**	11.08	C_28_H_32_O_16_	623.1605	−0.23	477.1034, 315.0507	Isorhamnetin-*O*-deoxyhexosyl-hexoside	x	x	x	x	[29]
**11**	11.22	C_33_H_40_O_19_	739.2083	0.41	593.1508, 285.0398	Kaempferol-*O*-dideoxyhexosyl-hexoside	x	x	x	x	[29]
**12**	11.30	C_38_H_48_O_23_	871.2501	−0.16	725.1930, 563.1384, 430.0899, 285.0396	Kaempferol-*O*-dideoxyhexosyl-pentosyl-hexoside	x	x	x	x	Figure S1
**13**	11.55	C_21_H_20_O_10_	431.1111	−4.19	285.0544	Kaempferol-*O*-deoxyhexoside	x	x	x	x	[22]
**14**	11.56	C_32_H_38_O_18_	709.1965	−1.31	563.1395, 431.0896, 285.0319	Kaempferol-*O*-dideoxyhexosyl-pentoside	x	x	x	x	[29]
**15**	11.74 *	C_27_H_30_O_14_	577.1682	−4.53	431.0971, 285.0544	Kaempferitrin	x	x	x	x	[22]
**16**	11.80	C_15_H_21_O_9_NS_2_	422.0577	0.67	274.9900, 259.0120, 195.0327, 96.9587, 74.9895	Gluconasturtiin	x	x	x	-	[30]
**17**	11.87	C_28_H_32_O_15_	607.1658	0.14	461.1079, 315.0505	Isorhamnetin *O*-dideoxyhexoside	x	x	x	x	[29]
**18**	12.48	C_16_H_22_O_8_	341.1236	1.39	179.0702	Coniferyl alcohol-*O*-deoxyhexoside	x	x	x	x	[31]
**19**	14.11	C_9_H_16_O_4_	187.0965	0.31	169.0856, 125.0958	Azelaic acid	x	x	x	x	[32]
**20**	15.34	C_21_H_20_O_10_	431.1118	0.65	285.0545	Kaempferol-*O*-deoxyhexoside	x	x	x	x	[22]
**21**	16.29	C_18_H_34_O_5_	329.2329	2.09	311.2229, 293.2111, 229.1441, 201.1121, 171.1029	9,12,13-Trihydroxyoctadecenoic acid	x	x	x	x	[20]
**22**	22.17	C_17_H_26_O_4_	293.1756	2.20	236.1047, 221.1539, 205.1213	6-gingerol	x	x	x	x	[20]
**23**	26.27	C_24_H_50_O_7_NP	540.3293 ^#^	−2.97	480.3075, 255.2326, 224.0645, 184.1734	Lyso-phosphatidylcholine (16:0)	x	x	x	x	[20]
**24**	31.70	C_16_H_32_O_3_	271.2273	2.06	225.2221, 75.0125	2-Hydroxyhexadecanoic acid	x	x	x	x	[24]
**25**	33.01	C_16_H_32_O_3_	271.2272	1.58	253.2235, 225.2221, 59.0129	3-Hydroxyhexadecanoic acid	x	x	x	x	[24]
**26**	33.87 **	C_19_H_36_O_3_	311.2585	1.31	267.2687, 239.2366, 71.0122	3-Hydroxynonadecenoic acid	x	x	x	x	[34]
**27**	34.39 **	C_19_H_38_O_3_	313.2740	0.87	283.2639, 75.0075	2-Hydroxynonadecanoic acid	x	x	x	x	[36]
**28**	34.95	C_18_H_36_O_3_	299.2583	0.63	253.2542, 59.0123	3-Hydroxyoctadecanoic acid	x	x	x	x	[34]
**29**	35.82 **	C_19_H_38_O_3_	313.2740	0.95	283.2692, 59.0073	3-Hydroxynonadecanoic acid	x	x	x	x	[36]
**30**	36.39 **	C_20_H_40_O_3_	327.2894	−0.01	309.0127, 116.5926, 59.0125	3-Hydroxyeicosanoic acid	x	x	x	x	[34]

The bold formatting was used to indicate the numbers corresponding to the compounds identified through LC-ESI/HRMS/MS analysis. F: flowers extract; L: leaves extract; S: stems extract; Fr: immature fruits extract; ^#^ related to [(M + FA) − H]^−^; R*_t_* related to flowers extract; * R*_t_* related to fruits extract; ** R*_t_* related to leaves extract. The symbol ‘x’ indicates that the compound was detected in the extract, whereas the symbol ‘–’ indicates that the compound was not detected.

With reference to the two main classes of specialized metabolites—namely glucosinolates and glycosylated flavonoids—the evaluation of the relative signal intensities of the individual metabolites across the different extracts indicated that glucosinolates were more abundant in the flowers compared to the other plant matrices (Table S1, Supplementary Materials). Glycosylated flavonoids were present at nearly comparable levels in all extracts, with the exception of the extract obtained from the stems.

The analysis of the existing literature highlights the presence of only a few studies aimed at assessing the chemical composition of *R. alba*. Among them, Soliman et al. [26] identified in the aerial parts of *R. alba* from Egypt nine flavonoids, such as quercetin, luteolin, kaempferol, rutin, kaempferol 3-*O*-rhamnoside, luteolin 7-*O*-rhamnoside, luteolin 7-*O*-glucoside, luteolin 7-*O*-(6″*O*-E-*p*-coumaroyl)-β-d-glucopyranoside, and kaempferol-3-*O*-β-d-glucopyranosyl-(1‴-2″)-*O*-α-l-rhamnopyranoside, and three phenolic acids such as *p*-hydroxy benzoic acid, chlorogenic acid, and *p*-coumaric acid. 

More recently, caffeic acid, protocatechuic acid, syringic acid, ferulic acid, rosmarinic acid, 6,7-dihydroxycoumarin, coumarin, rutin, and luteolin were identified in *R. alba* aerial parts collected in Algeria [16]. The aqueous extract of the aerial parts of *R. alba* collected in Morocco showed the presence of caffeic acid, *p*-coumaric acid, benzoic acid, gallic acid, vanillic acid, catechin, syringic acid, vanillin, quercetin, and rutin [17].

Ðulovíc et al. [5] analyzed the glucosinolate profiles of *R. alba* from Croatia. In *R. alba* flower, leaf, stem, and root, five glucosinolates, such as glucoconringiin, gluconasturtiin, glucobrassicin, neoglucobrassicin, and isobutyl glucosinolate, were identified. Gluconasturtiin was not found in the flower. In contrast, the chemical profiles of some *Reseda* species, such as *R. arabica*, *R. luteola*, *R. lutea*, *R. odorata*, *R. muricata*, *R. phyteuma*, and *R. villosa*, have been investigated [5,35,36,37,38,39,40,41,42,43,44,45].

Pagnotta et al. [46] investigated the presence of glucosinolates in *R. lutea* considering their accumulation pattern and profile during flowering time. The uncommon 2-(α-l-rhamnopyranosyloxy)benzyl glucosinolate, identified as the main glucosinolate in *R. lutea*, reached its highest content in the racemes during the full flowering stage. Its content decreased during late flowering, when the presence of 3-hydroxybenzyl glucosinolate increased.

### 2.2. Cell Viability

To offer an evidence-based framework for the potential safe and beneficial application of *R. alba*, the potential cytotoxic effects on normal (HFF1) and cancer (MCF-7, CaCo-2 and HepG-2) cells were herein investigated by measuring cell viability. None of the tested extracts affected the viability of HFF1 cells, used to test the safety in normal cells. Among species of the genus *Reseda*, several have been reported to possess relevant biological activities, including antitumor potential. In particular, *R. lutea* has been shown to exert anti-proliferative effects on different tumor cell lines, an activity generally attributed to its rich phytochemical profile, especially in flavonoids and other phenolic compounds [13,27]. However, no previous data were available regarding the antitumor properties of *R. alba*.

**Figure 2:**
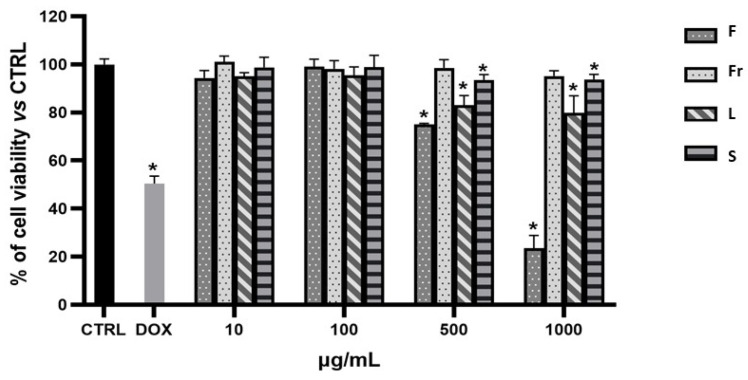
Cell viability assessed by MTT assay in MCF-7 cells: untreated control (CTRL), treated with doxorubicin (DOX) (positive control), and treated with various concentrations of *R. alba* extracts (F: flowers; L: leaves; S: stems; Fr: immature fruits) (10, 100, 500, and 1000 µg/mL) for 72 h. Values are the mean ± standard deviation (SD) of five experiments in triplicate. * Significant vs. untreated control cells: *p* ≤ 0.001.

In the present study, the methanol extracts of *R. alba* did not significantly affect the viability of MCF-7 breast cancer cells after 72 h of treatment, except at the highest concentrations tested (500–1000 μg/mL) (Figure 2).

**Figure 3:**
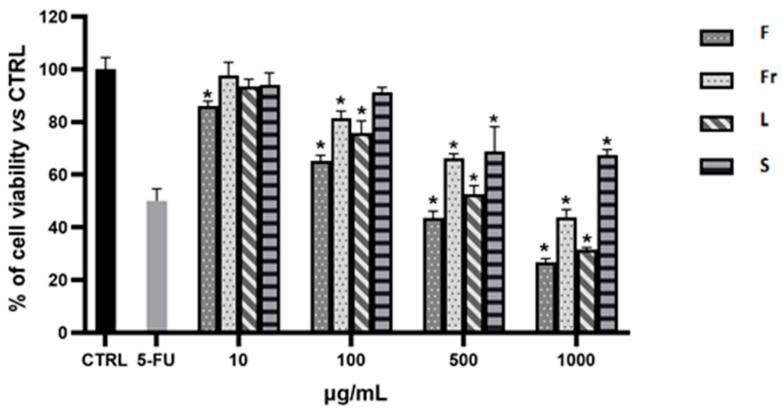
Cell viability assessed by MTT assay in CaCo-2 cells: untreated control (CTRL), treated with 5-fluorouracil (5-FU) (positive control), and treated with various concentrations of *R. alba* extracts (F: flowers; L: leaves; S: stems; Fr: immature fruits) (10, 100, 500, and 1000 µg/mL) for 72 h. Values are the mean ± SD of five experiments in triplicate. * Significant vs. untreated control cells: *p* ≤ 0.001.

**Figure 4:**
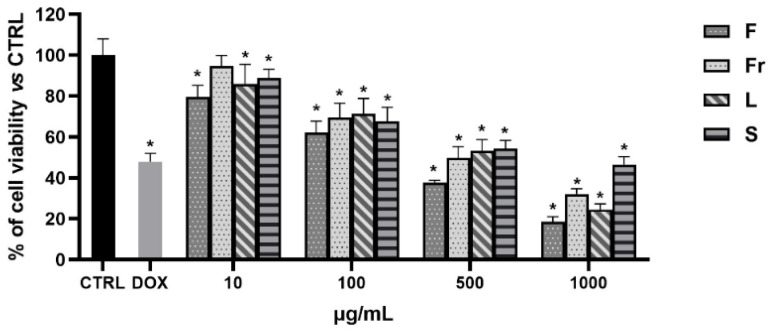
Cell viability assessed by MTT assay in HepG-2 cells: untreated control (CTRL), treated with DOX (positive control), and treated with various concentrations of *R. alba* extracts (F: flowers; L: leaves; S: stems; Fr: immature fruits) (10, 100, 500, and 1000 µg/mL) for 72 h. Values are the mean ± SD of five experiments in triplicate. * Significant vs. untreated control cells: *p* ≤ 0.001.

In contrast, the same treatments markedly reduced the viability of CaCo-2 and HepG-2 cells in a concentration-dependent manner, with effects detectable from 10 μg/mL, principally for the F and L extracts (Figure 3 and Figure 4). Particularly, F extract was the most effective, obtaining a decrease in cell viability of approximately 60% at 500 μg/mL, followed by L, Fr, and S extracts. No toxic effects were observed for any of the extracts after 24 h and 48 h of exposure. IC_50_ values for *R. alba* extracts, doxorubicin (DOX), and 5-fluorouracil (5-FU) are reported in Table 3.

This specific selective decrease in cell viability suggests a cell line-specific response, probably driven by intrinsic differences in cellular metabolism and drug resistance mechanisms [47]. The lower sensitivity observed in MCF-7 cells may be attributed to their well-documented capacity to develop resistance to xenobiotics, including plant-derived compounds [15]. This interpretation is consistent with previous findings demonstrating that CaCo-2 cells are more susceptible than MCF-7 cells to natural extracts [48]. Therefore, the differential response reported here likely represents a combination of pharmacodynamic factors and tumor-specific cellular characteristics.

The distinct suppression of cell viability seen across the tested cell lines may be related to differences in the qualitative phytochemical composition of the various *R. alba* extracts. Notably, the most active extracts (F and L) are characterized by the presence of specific metabolites, such as glucotropaeolin, which is exclusively detected in these fractions, as well as gluconasturtiin and glucochondringiin, which are also found in Fr and S extracts, as shown in Table 2.

Although *R. alba* remains poorly investigated, species within the genus *Reseda* are known to contain bioactive compounds, including phenolic acids, flavonoids, and glucosinolates [27]. These classes of metabolites are widely recognized for their roles in anti-cancer mechanisms, including inhibition of cell proliferation, induction of apoptosis, and modulation of oxidative stress [49].

In particular, glucosinolates and their hydrolysis products, such as isothiocyanates, have been widely investigated for their chemopreventive and antiproliferative properties. Consistently, glucotropaeolin, a benzyl glucosinolate, has been reported to exert growth-inhibitory effects against hepatocellular carcinoma and colon adenocarcinoma cell lines [50,51]. This evidence is particularly relevant to the present findings, as it supports the hypothesis that glucosinolate-derived compounds in *R. alba* may contribute to the observed reduced viability in CaCo-2 cells.

Furthermore, flavonoids identified in *R. alba* extracts, including kaempferol and isorhamnetin derivatives, are known to act through multiple molecular pathways, such as induction of cell cycle arrest and activation of apoptotic cascades. These effects are commonly mediated by the modulation of pro- and anti-apoptotic proteins (e.g., Bax and Bcl-2) and the activation of caspases [52,53].

Although these mechanisms were not directly investigated in the present study, they represent plausible pathways underlying the observed reduced cell viability. Overall, *R. alba* shows selective growth-inhibitory effects in colon cancer cells, supporting its potential as a source of bioactive compounds. Further studies are needed to identify the active metabolites and clarify their mechanisms of action.

### 2.3. Lactate Dehydrogenase (LDH) Release Quantification

Although there are many assays to detect apoptosis, relatively few tests are available to recognize necrosis. A key feature of necrotic cells is the permeabilization of the plasma membrane. This event can be quantified by measuring the release of lactate dehydrogenase (LDH), which is a cytoplasmic enzyme that catalyzes, during glycolysis, the interconversions of pyruvate to L-lactate and NADH to NAD+, and during the Cori cycle, the reverse reactions. Cellular injury, whether resulting from internal processes or external factors, can lead to the release of lactate dehydrogenase (LDH) from the cytoplasm into the surrounding medium.

Owing to its stability in culture conditions, LDH is widely utilized as a dependable biomarker for assessing cell and tissue damage, as well as cytotoxicity. Therefore, measuring LDH release is a useful method to detect necrosis [54].

**Figure 5:**
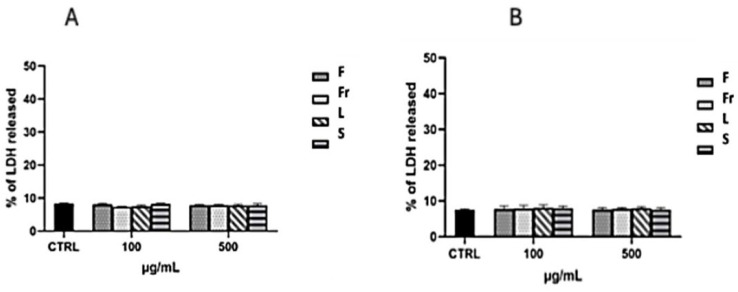
LDH release in CaCo-2 (**A**) and HepG-2 cells (**B**): untreated control (CTRL) and treated for 72 h with the *R. alba* extracts (100–500 μg/mL) (F: flowers; L: leaves; S: stems; Fr: immature fruits). Values are the mean ± SD of five experiments in triplicate.

In our study, the LDH release assay showed that the treatments with *R. alba* extracts did not induce the release of the marker enzyme in CaCo-2 and HepG-2 cells (Figure 5), allowing us to hypothesize the involvement of a different cell death pathway.

Numerous studies have shown that polyphenols can block anti-apoptotic factors and/or activate pro-apoptotic molecules [55].

A recent review reported apoptosis as a probable mechanism of cell death implicated in the anti-proliferative effects of *R. lutea* and *R. odorata* against various types of human cancers [56]. *R. luteola* extract has been shown to have anti-proliferative and pro-apoptotic effects on PHA-stimulated peripheral blood mononuclear cells. The author attributed this effect to the flavonoid fraction, particularly luteolin and its derivatives [57]. Our findings suggest that the growth-inhibitory effects of the different *R. alba* extracts on CaCo-2 cells are not related to necrotic cell death, but rather to a different type of cell death [58]. This activity appears to be linked to specific secondary metabolites, whose concentrations vary across the different fractions that together form a distinctive phytocomplex—particularly potent in the F extract.

### 2.4. Anti-Inflammatory Activity

Plants have long been recognized as an important reservoir of biologically active molecules whose beneficial effects in the prevention and/or treatment of different pathological conditions are mainly associated with their anti-inflammatory and antioxidant activities. In particular, phenolic compounds have emerged as promising agents due to their ability to modulate cellular pathways involved in oxidative stress and inflammation. These compounds can influence the activity and expression of several pro-inflammatory enzymes, including cyclooxygenase (COX), lipoxygenase (LOX), and nitric oxide synthase (NOS). NOS catalyzes the production of nitric oxide (NO), a key inflammatory mediator whose excessive production has been associated with chronic inflammation and tissue damage. Through the inhibition of these enzymatic pathways and the scavenging of reactive oxygen species, phenols may exert protective effects against inflammation-related disorders [59].

**Figure 6:**
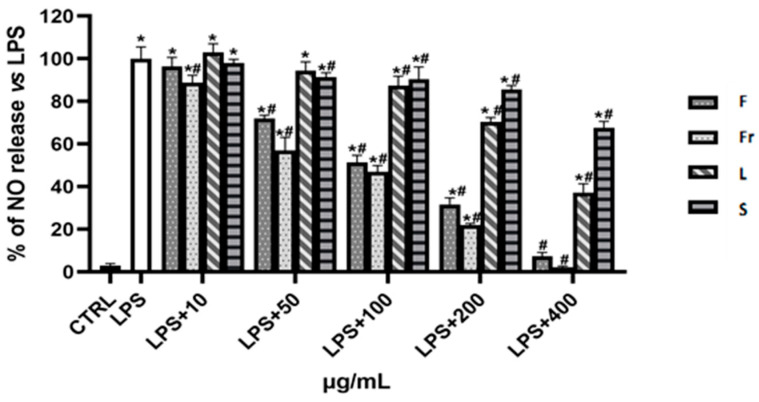
S: stems; Fr: immature fruits; F: flowers; L: Leaves. RAW 264.7 untreated cells (CTRL), LPS-treated for 2 h, and pre-treated with the *R. alba* extracts (10–50–100–200–400 μg/mL) for 24 h. Values are the mean ± SD of five experiments in triplicate. * Significant vs. LPS-treated cells: *p* < 0.001. ^#^ Significant vs. untreated control cells: *p* ≤ 0.001.

Herein, the investigated extracts showed NO inhibitory activity on RAW 264.7 cells as an in vitro model of LPS-induced inflammation. Notably, Fr extract elicited the highest effect, inhibiting NO production by 50% at the concentration of 62.50 µg/mL in LPS-activated RAW 264.7 cells (Figure 6).

The remaining extracts (F, L, and S) also exhibited inhibitory activity, although to a lesser extent, with S and L extracts generally showing moderate effects at intermediate and higher concentrations. Overall, these results suggest that all extracts possess anti-inflammatory potential, with Fr extract being the most effective in suppressing LPS-induced NO production in macrophages.

The highest anti-inflammatory activity exerted by Fr extract is likely associated with its greater flavonoid content compared to the other extracts. These findings, together with the lack of toxicity of the different extracts on human fibroblasts and murine macrophages, support the pharmacological activity of *R. alba* extract in the treatment of inflammatory skin disorders.

Previous phytochemical studies on *Reseda* species, especially on *R. luteola*, have highlighted a rich profile of phenolic constituents, principally flavonoids, including luteolin-7-*O*-glucoside, apigenin-7-*O*-glucoside, and several quercetin and kaempferol derivatives. These flavonoids are reported as promising anti-inflammatory compounds [60]. Luteolin, a flavonoid identified in *R. odorata*, has been reported to display a concentration-dependent anti-inflammatory activity in macrophages through the inhibition of NO production [56].

### 2.5. Antioxidant Properties

The antioxidant efficacy of a compound depends on several factors, including its chemical structure, concentration, reaction kinetics, temperature, substrate characteristics, and the presence of synergistic or pro-oxidant compounds [61]. For these reasons, herein, the antioxidant activity of *R. alba* extracts was evaluated using a multi-target approach by applying different tests such as ABTS, FRAP, and β-carotene bleaching assays. Free radical scavenging activity of the *R. alba* extracts was evaluated using the ABTS test. FRAP test was used to quantify the ferric reducing antioxidant power, while the β-carotene bleaching test was employed to investigate the ability of *R. alba* extracts to inhibit lipid peroxidation. Results are reported in Table 4.

The relative antioxidant activity of *R. alba* extracts to scavenge the radical ABTS^+^ has been compared with ascorbic acid, used as a positive control (IC_50_ of 1.7 μg/mL). In this assay, the most promising extracts, which exhibited the highest total phenolic and flavonoid contents, are Fr and S with IC_50_ values of 3.9 and 4.0 μg/mL, respectively.

The most active extract in inhibiting lipid peroxidation is the extract of leaves with an IC_50_ of 37.6 μg/mL after 30 min of incubation.

A comparable activity was observed for S and Fr extracts. In the FRAP test, values in the range of 16.7–39.5 μM Fe (II)/g were found. The most active sample was the extract of *R. alba* stems (39.5 μM Fe (II)/g), followed by flowers and leaves (21.7 μM Fe (II)/g).

The available literature shows that the antioxidant activity of only three *Reseda* species, namely *R. alba*, *R. lutea*, and *R. muricata*, has been investigated to date. These species exhibited noteworthy antioxidant properties, although this activity strongly depends on the extraction solvent and plant material used. Table 5 summarizes data reported in the literature on the antioxidant properties of *Reseda* species.

The antioxidant activity of *R. lutea* ethanol and aqueous extracts, explored by DPPH, ABTS, Fe^2+^ chelating, Fe^3+^ reduction, CUPRAC, and FRAP tests [15], revealed a generally moderate antioxidant activity. Differences depending on both the extraction solvent and the test used are evident. In particular, the ethanol extract showed higher radical scavenging activity compared to the aqueous one.

The extracts of *R. alba* [16] showed more evident antioxidant effects compared to *R. lutea* and *R. muricata*, especially in ABTS and FRAP tests. Among the tested extracts, the ethyl acetate fraction of *R. alba* exhibited remarkable ABTS scavenging activity with an IC_50_ value of 13.57 µg/mL, while the dichloromethane extract displayed the strongest reducing power in the FRAP assay with an A_0.50_ value of 4.30 µg/mL.

Nacer et al. [16] investigated extracts from whole aerial parts of *R. alba*. In contrast, the present study provides, for the first time, a comparative evaluation of extracts obtained from different parts of this species, such as leaves, flowers, stems, and immature fruits. This organ-specific approach allowed the identification of differences in both chemical profile and biological effects, highlighting the contribution of individual plant organs to the biological potential of this species.

## 3. Materials and Methods

### 3.1. Plant Materials

**Figure 7:**
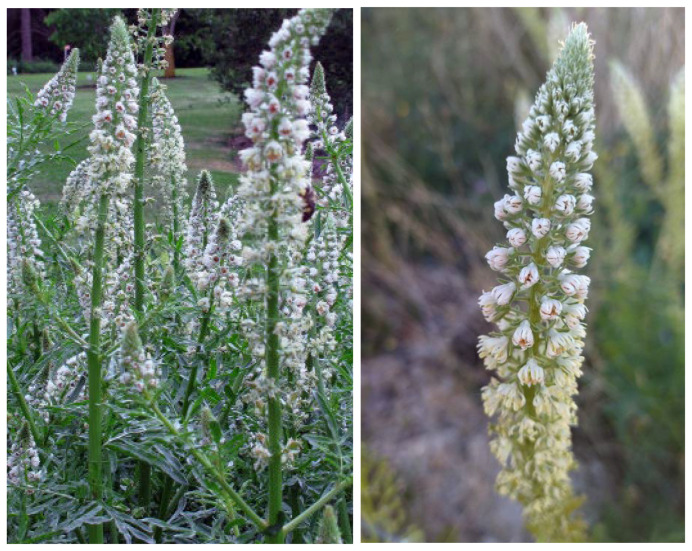
*Reseda alba* L. (Resedaceae) from Calabria, Southern Italy.

Flowers, leaves, stems, and immature fruits of *R. alba* (Figure 7) were randomly harvested from 10 plants in the full flowering stage in April 2024 in Calabria (San Lucido, Cosenza, Southern Italy, along the coastal road between San Lucido and Paola, railway embankment, 2 m; WGS84: 39.318594° N, 16.044151° E) by Prof. Nicodemo G. Passalacqua, who botanically identified the species.

Before extraction, the plant materials were checked to verify the integrity and absence of contamination.

A voucher specimen is deposited in the Herbarium of the University of Calabria under the registration number n. CLU 26306. Before extraction, samples were checked to verify the integrity and absence of contamination.

### 3.2. Chemicals and Reagents

Solvents (methanol, water, and acetonitrile) were purchased from VWR International s.r.l. (Milan, Italy). Solvents for LC-ESI/HRMS/MS analysis (methanol, water, acetonitrile, formic acid) were purchased from Merck (Milan, Italy). Chlorogenic acid, quercetin, β-carotene, ascorbic acid, 2,2′-azino-bis(3-ethylbenzothiazoline-6-sulfonic acid (ABTS) solution, aluminum chloride solution, formic acid, Folin–Ciocalteau reagent, sodium carbonate, 3-(4,5-dimethylthiazol-2-yl)-2,5-diphenyl tetrazolium bromide (MTT), butylated hydroxytoluene (BHT), iron(III) chloride solution, 2,2-diphenyl-1-picrylhydrazyl (DPPH), Tween 20, linoleic acid, dimethyl sulfoxide (DMSO), Dulbecco’s Modified Eagle Medium (DMEM), propyl gallate, Griess reagent, β-nicotinamide adenine dinucleotide, reduced disodium salt (NADH), lipopolysaccharides (LPS), doxorubicin, and 5-fluorouracil were purchased from Sigma-Aldrich S.p.a. (Milan, Italy).

### 3.3. Sample Preparation

The fresh flowers (250 g), leaves (250 g), stems (250 g), and immature fruits (120 g) of *R. alba* were subjected to ultrasound-assisted extraction (UAE) by using methanol as solvent. A Branson 3800 ultrasonic system, series CPXH (130 W, 40 kHz frequency) (Milan, Italy), was used. For each sample, three cycles were done, each of 1 h (3 × 900 mL for flowers, leaves, and stems; 3 × 500 mL for immature fruits).

At the end of the extraction procedure, solutions were filtered and evaporated using a rotary evaporator at 35 °C in order to obtain the total extract. Extracts were stored in brown glass bottles, hermetically sealed, and kept at −20 °C until chemical and biological assays.

### 3.4. Total Phenolic, Flavonoid, and Carotenoid Content

The Total Phenolic Content (TPC) was assessed by using the Folin–Ciocalteau method [63], based on reducing tungstate and/or molybdate in the Folin–Ciocalteau reagent by phenols in an alkaline medium, resulting in a blue-colored solution. In brief, the extract at a concentration of 1.5 mg/mL was mixed with 0.2 mL of Folin–Ciocalteau reagent, 2 mL of water, and 1 mL of 15% sodium carbonate. After incubation at 25 °C for 2 h, the absorbance was read at 765 nm by using a UV−vis Jenway 6003 spectrophotometer (Milan, Italy). Chlorogenic acid was used as a standard for comparison, and the results are expressed in milligrams of chlorogenic acid equivalents/g of plant material. All determinations were performed in triplicate.

The Total Flavonoid Content (TFC) was determined using the technique based on the formation of a coordination complex involving the Al^3+^ cation [61]. This assay enables the quantification of the Total Flavonoid Content present in the sample; however, it does not permit the identification or characterization of individual flavonoid compounds. It is also unsuitable for the determination of certain flavonoid subtypes, such as isoflavones, where specific ring substitutions do not allow for complexation with Al^3+^. The sample at a concentration of 1.5 mg/mL was mixed with 2% aluminum chloride solution and incubated at 25 °C. After 15 min, the absorbance was measured at 510 nm. Quercetin was used as a standard for comparison, and the results are expressed in milligrams of quercetin equivalents/g of plant materials. All determinations were performed in triplicate.

The Total Carotenoid Content (TCC) was determined as previously described [61]. Briefly, 1 mL of the extract at a concentration of 1.5 mg/mL was added to 0.5 mL of NaCl 5% solution, vortexed for 30 s and centrifuged at 4500 rpm for 10 min. After dilution with *n*-hexane, the absorbance of the supernatant was read at 460 nm. β-Carotene was used as a standard for comparison, and the results are expressed in milligrams of β-carotene equivalent/g of plant materials. All determinations were performed in triplicate.

### 3.5. LC-ESI/HRMS/MS Analysis

Qualitative analysis using UHPLC–(−)ESI–Q Exactive MS/MS was carried out using an ultra-high-performance liquid chromatography system (UltiMate 3000, Dionex, Sunnyvale, CA, USA) coupled to an electrospray ionization source and a high-resolution Q Exactive mass spectrometer (Thermo Fisher Scientific, Waltham, MA, USA). The extracts were prepared in methanol–water (1:1, *v*/*v*) at a final concentration of 1.0 mg/mL, and a 5 μL aliquot was injected for LC-HRMS analysis.

The mass analyzer was calibrated in accordance with the manufacturer’s guidelines using a standard calibration solution containing sodium dodecyl sulfate, sodium taurocholate, and Ultramark 1621. Data acquisition and processing were performed using the manufacturer’s proprietary software, as previously described [64,65].

Chromatographic separation was achieved on a Luna C18 column (5 μm, 150 × 2.1 mm; Phenomenex, Milan, Italy) operated at a flow rate of 0.2 mL/min. The mobile phase consisted of water with 0.1% formic acid (solvent A) and acetonitrile with 0.1% formic acid (solvent B). The gradient program began at 5% B, increased linearly to 95% over 35 min and was held at this % for 5 min before returning to initial conditions within 5 min. The ESI source was operated with a capillary voltage of −48 V, a tube lens voltage of −176.47 V, and an ion source temperature of 280 °C. Nitrogen was used as the sheath and auxiliary gas at flow rates of 15 and 5, respectively.

Full-scan MS data were acquired over an *m*/*z* range of 120–1400. MS/MS experiments were conducted in data-dependent acquisition mode, in which the most intense precursor ions were selected for fragmentation using a normalized collision energy of 30%. Instrument control, data acquisition, and data analysis were performed using Xcalibur software.

### 3.6. Cell Cultures

Human foreskin fibroblast cell line HFF-1 (SCRC-1041, ATCC^®^, Rockville, MD, USA) and mouse leukemic monocyte-macrophage cells RAW 264.7 (91062702, Sigma-Aldrich Milan, Italy) were grown in Dulbecco’s Modified Eagle Medium (DMEM) containing 10% *v*/*v* FBS, 1 mmol/L sodium pyruvate, 4 mM L-glutamine, streptomycin (10 μg/mL), and penicillin (100 U/mL). The human breast cancer cell line MCF-7 (HTB-22, ATCC^®^, Rockville, MD, USA), human colon carcinoma cells CaCo-2 (HTB-37, ATCC^®^, Rockville, MD, USA) and human hepatoma cell line HepG-2 (HTB-37, ATCC^®^, Rockville, MD, USA), were cultured in MEM supplemented with 10% *v*/*v* FBS, 1 mmol/L sodium pyruvate, 2 mmol/L L-glutamine, streptomycin (10 μg/mL), penicillin (100 U/mL), and 1% non-essential amino acids. To ensure consistent experimental conditions across all experiments, cell lines were plated at a constant density of 8 × 10^3^ cells/well and kept at 37 °C in an incubator humidified with 5% CO_2_. HFF-1 cells were employed as a human model for preliminary toxicity screening; RAW 264.7 cells were used as an in vitro model of LPS-induced inflammation, while MCF-7, CaCo-2 and HepG-2 cells were used as in vitro preliminary screens of anti-cancer activities.

### 3.7. MTT Test

*R. alba* extracts at concentrations of 10, 100, 500, and 1000 μg/mL were applied to HFF-1, RAW 264.7, MCF-7, CaCo-2 and HepG-2 cell lines for 24, 48, and 72 h. The MTT assay measures a cell’s capacity to metabolize 3-(4,5-dimethylthiazol-2-yl)-2,5-diphenyl tetrazolium bromide (MTT) using mitochondrial succinic dehydrogenase. Once inside the cells, MTT reaches the mitochondria and is converted into formazan, an insoluble colored product [48]. The quantity of live cells is commensurate with the amount of formazan. With a microplate spectrophotometer reader (Titertek Multiskan, Flow Laboratories, Helsinki, Finland), the absorbance of the converted formazan was determined at 570 nm. Doxorubicin and 5-fluorouracil were used as reference compounds in MCF-7, HepG-2 and CaCo-2, respectively. Wells containing only culture medium (without cells) were used as a blank control to account for background absorbance. The average absorbance value of these blank wells was subtracted from the optical density values of all experimental wells before calculating cell viability. Results are expressed as percentage of cell viability compared with untreated control cells and presented as the mean ± SD of four independent experiments.

### 3.8. Lactate Dehydrogenase (LDH) Release Assay

To evaluate cell membrane integrity and quantify cell necrosis resulting from membrane rupture, a lactate dehydrogenase (LDH) release assay was performed. CaCo-2 and HepG-2 cells were seeded in 24-well plates at 1 × 10^5^ cells/well and, after 24 h, treated with *R. alba* extracts at 100 and 500 µg/mL for 72 h. After incubation, the culture medium was collected, and cells were lysed for 1 h with a digitonin solution (2.5 mg/mL) to estimate the released and intracellular LDH, respectively. Specifically, LDH activity was measured spectrophotometrically at 340 nm by monitoring the decrease in NADH absorbance, which is directly proportional to enzyme activity. The reaction mixture consisted of sodium pyruvate (1.0 mM), NADH (0.2 mM), and biological sample (30 µL of cell lysate or 100 µL of cell medium) in potassium phosphate buffer (100 mM, pH 7.5).

The total LDH amount was defined as the sum of the enzyme activity measured in both the culture medium and the cell lysate. The percentage of released LDH was calculated as the *ratio* of extracellular LDH activity to total LDH activity. Data are reported as the percentage of total LDH released ± SD of three independent experiments.

### 3.9. Nitric Oxide (NO) Inhibitory Activity

The inhibitory effect of *R. alba* extracts on NO generation was evaluated by measuring the amount of nitrite using Griess reagent [63]. LPS (2 µg/mL) was used to activate RAW 264.7 cells for two hours following a 24 h pre-treatment with distinct concentrations of *R. alba* extracts (10, 50, 100, 200, and 400 µg/mL). In short, following the treatments, the reaction mixture composed of 250 µL of culture media and 250 µL of Griess reagent was incubated for 10 min at room temperature [63]. Griess reagent is used in the experiment to assess the nitrite diazo-coupling reaction. Using a Synergy HT plate reader (BioTek Instruments, Inc., Winooski, VT, USA), the amount of nitrite in the culture medium was measured at 540 nm.

### 3.10. Antioxidant Assays

A number of assays have been introduced to measure the antioxidant activity of plant-based phytochemicals. The antioxidant activity depends on their chemical structure. Specifically, it depends on their ability to delocalize the unpaired electron within the aromatic structure, to donate hydrogen with electron, and metal chelation.

Herein, *R. alba* extracts have been subjected to different assays to study their potential antioxidant activity, such as the ABTS test, FRAP test, and β-carotene bleaching test.

In the ABTS assay, the pre-formed radical monocation of 2,2′-azinobis-(3-ethylbenzothiazoline-6-sulfonic acid) is generated by oxidation of ABTS with potassium persulfate and is reduced in the presence of such hydrogen-donating antioxidants. In short, a solution of the ABTS radical cation was prepared by mixing ABTS solution 7 mM with potassium persulfate 2.45 mM and stored at room temperature in the dark. After 12 h, a mixture of the sample (at concentrations in the range 1–400 µg/mL) and diluted ABTS solution was prepared. After 6 min, the absorbance was read at 734 nm. Ascorbic acid was used as the positive control. Data are reported as IC_50_ values ± SD of three independent experiments [63].

In the FRAP test, FRAP reagent was prepared by mixing tripyridyltriazine (TPTZ) solution 10 mM with HCl 40 mM, acetate buffer (pH 3.6) and iron(III) chloride 20 mM [63]. A mixture of sample (at a concentration of 2.5 mg/mL), water, and FRAP reagent was prepared and incubated for 30 min at 25 °C. The absorbance was measured at 595 nm. The value was. Butylated hydroxytoluene (BHT) was used as a positive control. Data are expressed as μM Fe(II)/g ± SD of three independent experiments.

The protection of lipid peroxidation was tested by applying the β-carotene bleaching assay. As previously described, an emulsion containing β-carotene, Tween 20, and linoleic acid was mixed with the samples at concentrations in the range of 5–100 μg/mL [63]. The absorbance was read at 470 nm after 30 min of incubation at 45 °C. Propyl gallate was used as a positive control. Data are reported as IC_50_ values ± SD of three independent experiments.

### 3.11. Statistical Analysis

Data are expressed as means ± standard deviations (SDs). GraphPad Prism 4.0 Software (San Diego, CA, USA) was used to calculate the concentration giving 50% inhibition (IC_50_). In both antioxidant assays and enzyme inhibition tests, differences within and between groups were evaluated by ANOVA followed by the Tukey test. In cell culture-based tests, differences between means were analyzed by Student’s *t*-test.

## 4. Conclusions

Although significant progress has been made in the development of novel pharmaceutical agents, natural sources continue to provide a rich reservoir of structurally diverse and biologically active compounds with relevant therapeutic potential. In particular, natural products play a crucial role in the discovery of new lead compounds for the prevention and treatment of complex diseases, including cancer and chronic inflammatory disorders, which remain major global health challenges. In this context, *R. alba* has appeared as a promising candidate for health-related applications.

The present paper offers the first in-depth study of the biological potential of *R. alba* extracts and their fractions as potential anti-inflammatory, antioxidant and growth-inhibitory agents, emphasizing organ-specific activities and showing novel biological insights. This organ-specific approach allowed the identification of marked differences in both chemical composition and biological activities, highlighting the contribution of individual plant organs to the overall biological potential of the species. Overall, the flower extract demonstrated the highest cytostatic activity against CaCo-2 colorectal cancer cells and HepG-2 hepatocellular carcinoma cells.

On the other hand, among the extracts tested for their potential anti-inflammatory properties using an in vitro model of LPS-induced inflammation with RAW 264.7 macrophages, the Fr extract showed the most significant activity, reducing NO production by 50% at a concentration of 62.50 µg/mL. This enhanced effect is likely related to its higher flavonoid content, suggesting a key role of these compounds in modulating inflammatory responses. Importantly, no cytotoxic effects on human fibroblasts and murine macrophages were found, supporting their safety profile and potential suitability for topical applications. The antioxidant potential of *R. alba* extracts was demonstrated through a multi-target analytical approach, including ABTS radical scavenging, FRAP reducing power, and β-carotene bleaching assays. The results revealed that the extracts possess significant antioxidant activity, although with different effectiveness depending on the plant part and the assay applied. Among the tested samples, the Fr and S extracts exhibited the strongest ABTS radical scavenging activity. Conversely, the L extract showed the highest ability to inhibit lipid peroxidation after 30 min of incubation.

These results highlight the biological potential of *R. alba* extracts, particularly the F and Fr extracts. Further studies are warranted to elucidate the molecular mechanisms involved and to evaluate their efficacy in in vivo models.

## Figures and Tables

**Table 1:** Extraction yield and total phytochemical content of *R. alba* extracts.

*R. alba*	Extraction Yield (%)	TPC ^1^	TFC ^2^	TCC ^3^
Stems (S)	9.9 ^b^	20.1 ± 1.2 ^b^	9.8 ± 1.2 ^b^	5.6 ± 0.4 ^a^
Immature fruits (Fr)	5.3 ^c^	30.9 ± 1.7 ^a^	17.7 ± 0.5 ^a^	3.1 ± 0.2 ^b^
Flowers (F)	12.2 ^a^	10.2 ± 1.4 ^d^	7.8 ± 0.3 ^c^	2.6 ± 0.2 ^b^
Leaves (L)	11.7 ^a^	15.3 ± 0.8 ^c^	9.1 ± 0.5 ^b^	6.1 ± 0.3 ^a^
*Sign.*	*	**	*	*

Values are the means of three experiments ± standard deviation (SD). ^1^ TPC: Total Phenolic Content, mg equivalents of chlorogenic acid/g of plant materials. ^2^ TFC: Total Flavonoid Content, mg equivalents of quercetin/g of plant materials. ^3^ TCC: Total Carotenoid Content, mg equivalents of β-carotene/g of plant materials. Statistical analyses using ANOVA were followed by Tukey’s post hoc test. Results followed by letters are significant at ** *p* ≤ 0.01; * *p* ≤ 0.05.

**Table 3:** IC_50_ values (concentration that inhibits 50% of the cell viability, μg/mL) at 72 h of *R. alba* extracts and positive control (doxorubicin, DOX: MCF-7, HepG-2; 5-fluorouracil, 5-FU: CaCo-2) on three cancer cell lines.

Cell Line	*R. alba* Extracts				Positive Control
	F	Fr	L	S	
MCF-7	N.D. ^a^	N.D. ^a^	N.D. ^a^	N.D. ^a^	19.0 ± 1.5
CaCo-2	326 ± 20	850 ± 22	608 ± 18	N.D. ^a^	10.0 ± 1.0
HepG-2	390.30 ± 16	592.89± 19	541.39 ± 11	764.87 ± 22	0.85 ± 0.1

F: flowers; L: leaves; S: stems; Fr: immature fruits. Values are the mean ± SD of five experiments in triplicate. ^a^ not determinable.

**Table 4:** Antioxidant activity of *R. alba* extracts.

*R. alba* Extract	FRAP Test μM Fe (II)/g ^a^	ABTS Test IC_50_ (μg/mL)	β-Carotene Bleaching Test IC_50_ (μg/mL)	
			30 min	60 min
Stems (S)	39.5 ± 1.8 ^a^	4.0 ± 0.5 ^a^	71.7 ± 2.2 ^b^	91.6 ± 2.8 ^b^
Immature fruits (Fr)	16.7 ± 1.0 ^c^	3.9 ± 0.4 ^a^	72.2 ± 2.5 ^b^	85.6 ± 2.6 ^a^
Flowers (F)	21.7 ± 1.5 ^b^	21.3 ± 0.9 ^c^	>100	>100
Leaves (L)	21.7 ± 1.1 ^b^	5.5 ± 0.6 ^b^	37.6 ± 1.0 ^a^	>100
*Sign.*	**	**	*	*

Values are the means of three experiments ± SD. Ascorbic acid was used as a positive control in ABTS (IC_50_ 1.7 μg/mL), propyl gallate in β-carotene bleaching test (IC_50_ 0.09 and 1.0 μg/mL after 30 and 60 min of incubation), and butylhydroxytoluene (BHT) in the FRAP test (IC_50_ 63.3 μg/mL). Statistical analyses using ANOVA were followed by Tukey’s post hoc test. Results with superscript letters are significant at ** *p* ≤ 0.01; * *p* ≤ 0.05.

**Table 5:** Data reported in the literature on antioxidant effects of *Reseda* species.

Species	Plant Part	Extract	Test	Activity IC_50_ (µg/mL)	Reference
*R. alba*	aerial parts	Dichloromethane	DPPH	26.16	[16]
		Ethyl acetate	DPPH	88.66	[16]
		*n*-Butanol	DPPH	72.14	[16]
		Dichloromethane	ABTS	31.33	[16]
		Ethyl acetate	ABTS	13.57	[16]
		*n*-Butanol	ABTS	22.74	[16]
		Dichloromethane	FRAP	4.30 ^a^	[16]
		Ethyl acetate	FRAP	37.77 ^a^	[16]
		*n*-Butanol	FRAP	94.33 ^a^	[16]
		Dichloromethane	CUPRAC	78.22 ^a^	[16]
		Ethyl acetate	CUPRAC	86.59 ^a^	[16]
		*n*-Butanol	CUPRAC	69.70 ^a^	[16]
		Dichloromethane	GOR	50.12	[16]
		Ethyl acetate	GOR	47.76	[16]
		*n*-Butanol	GOR	38.47	[16]
*R. lutea*	aerial parts	Ethanol	DPPH	231.0	[15]
		Aqueous	DPPH	346.50	[15]
	flowers	Aqueous	DPPH	13.4% ^b^	[15]
*R. muricata*	aerial parts	Methanol	DPPH	154.80	[62]

GOR: Galvinoxyl radical; CUPRAC: CUPric Reducing Antioxidant Capacity; ^a^ A_0.50_: effective concentration producing an absorbance of 0.50 (µg/mL); ^b^ % of inhibition.

## Data Availability

The original contributions presented in this study are included in the article/Supplementary Material. Further inquiries can be directed to the corresponding author.

## Supplementary Materials

The following supporting information can be downloaded at: https://www.mdpi.com/article/10.3390/plants15121821/s1, Table S1: relative peak intensity of metabolites identified in *R. alba* extracts by LC-ESI/HRMS/MS analysis. Figure S1: ESI–Q Exactive MS/MS spectrum of compound 12 (A) and its corresponding MS/MS spectrum (B) in negative ion mode.

## Abbreviations

The following abbreviations are used in this manuscript:

A_0.50_	Concentration Producing an Absorbance of 0.50
ABTS	2,2-Azino-bis (3-ethylbenzothiazoline-6-sulfonic acid)
BHA	Butylated Hydroxyanisole
BHT	Butylhydroxytoluene
CAE	Chlorogenic Acid Equivalents
COX	Cyclooxygenase
CRC	Colorectal Cancer
CUPRAC	CUPric Reducing Antioxidant Capacity
DOX	Doxorubicin
DPPH	1,1-Diphenyl-2-picrylhydrazyl
DMEM	Dulbecco’s Modified Eagle Medium
5-FLU	5-Fluorouracil
FRAP	Ferric Reducing Antioxidant Power
HFF-1	Human Foreskin Fibroblast
IC_50_	Half Maximal Inhibitory Concentration
LC-ESI/HRMS/MS	Liquid Chromatography/Electrospray/High-Resolution Tandem–Mass Spectrometry
LDH	Lactic Dehydrogenase
LOX	Lipoxygenase
LPS	Lipopolysaccharide
MTT	3-(4,5-dimethylthiazol-2-yl)-2,5-diphenyl tetrazolium bromide
NO	Nitric Oxide
NOX	Nitric Oxide Synthase
PG	Propyl Gallate
PUFAs	Polyunsaturated Fatty Acids
QE	Quercetin Equivalents
SD	Standard Deviation
TCC	Total Carotenoid Content
TFC	Total Flavonoid Content
TPC	Total Phenolic Content
