# Supplementary material for: Chemical Profile and Evaluation of the Growth-Inhibitory, Anti-Inflammatory, and Antioxidant Activity Potential of Polar Extracts of Reseda alba L. (Resedaceae)
Source: Plants (Basel). 2026 Jun 12;15(12):1821. doi: 10.3390/plants15121821 (PMC13306858; doi:10.3390/plants15121821)
Supplement: Supplementary file 1 [file plants-15-01821-s001.zip › plants-4331637-supplementary.pdf]

plants

MDPI

Chemical Profile and Evaluation of the Growth-inhibitory, Anti-inflammatory, and Antioxidant Potential Activity of Polar Extracts of *Reseda alba* L. (Resedaceae)

Giuseppe A. Malfa, Antonietta Cerulli, Donata Condorelli, Assunta Napolitano, Elena Preite, Nicodemo G. Passalacqua, Monica R. Loizzo, Sonia Piacente, and Rosa Tundis

Supplementary Materials

Table S1. Relative peak intensity of metabolites identified in *R. alba* extracts by LC-ESI/HRMS/MS analysis.

n	t_R	Mol Formula	[M-H]-	Name	F	L	S	Fr
1	1.82	$C_{12}H_{22}O_{11}$	341.1079	Sucrose	$2.96x10^8$	$4.55x10^7$	$3.78x10^8$	$1.96x10^8$
2	2.34	$C_9H_{19}O_{11}P$	333.0586	Glycerophosphoryl inositol	$1.30x10^7$	$1.23x10^7$	$1.14x10^7$	$3.59x10^7$
3	3.21	$C_{11}H_{21}O_{10}NS_2$	390.0525	Glucoconringiin	$1.01X10^8$	$5.89x10^4$	---	$3.07x10^6$
4	7.45	$C_{11}H_{21}O_9NS_2$	374.0579	Methylpropyl glucosinolate	$6.40X10^6$	$3.93x10^5$	$3.29x10^4$	$3.79x10^5$
5	8.97*	$C_{27}H_{32}O_{15}$	595.1666	Naringenin-di-C-glucoside	$6.68X10^6$	$9.38x10^6$	$1.32x10^7$	$1.57x10^7$
6	9.51	$C_{14}H_{19}O_9NS_2$	408.0418	Glucotropaeolin	$6.70x10^4$	$2.06x10^5$	---	---
7	9.91	$C_{14}H_{18}O_9$	329.0873	Vanillic acid-*O*-hexoside	$1.04x10^5$	$6.77x10^5$	$9.33x10^6$	---
8	10.20	$C_{27}H_{30}O_{16}$	609.1452	Rutin	$1.17x10^7$	$3.23x10^7$	$1.36x10^7$	$1.66x10^7$
9	10.98	$C_{27}H_{30}O_{15}$	595.1495	Kaempferol 3-*O*-rutinoside	$2.61x10^8$	$7.60x10^7$	$2.28x10^7$	$1.80x10^8$
10	11.08	$C_{28}H_{32}O_{16}$	623.1605	Isorhamnetin-*O*- deoxyhexosyl-hexoside	$5.19x10^7$	$7.98x10^7$	$2.67x10^7$	$5.10x10^7$
11	11.22	$C_{33}H_{40}O_{19}$	739.2083	Kaempferol-*O*-di-deoxyhexosyl-hexoside	$2.38x10^7$	$2.75x10^7$	$9.27x10^6$	$2.74x10^7$
12	11.30	$C_{38}H_{48}O_{23}$	871.2501	Kaempferol- *O*-di-deoxyhexosyl-pentosyl-hexoside	$2.77x10^7$	$1.52x10^7$	$1.60x10^7$	$2.91x10^7$
13	11.55	$C_{21}H_{20}O_{10}$	431.1111	kaempferol-*O*-deoxyhexoside	$1.05x10^6$	$1.00x10^6$	$1.10x10^6$	$9.89x10^6$
14	11.56	$C_{32}H_{38}O_{18}$	709.1965	Kaempferol-*O*-dideoxyhexosyl-pentoside	$2.10x10^8$	$2.80x10^8$	$3.69x10^7$	$2.10x10^8$
15	11.74*	$C_{27}H_{30}O_{14}$	577.1682	Kaempferitrin	$2.67x10^8$	$3.52x10^7$	$1.45x10^7$	$1.46x10^8$
16	11.80	$C_{15}H_{21}O_9NS_2$	422.0577	Gluconasturtiin	$7.30x10^6$	$9.25x10^4$	$7.60x10^6$	---
17	11.87	$C_{28}H_{32}O_{15}$	607.1658	Isorhamnetin *O*-di-deoxyhexoside	$1.14x10^7$	$1.84x10^7$	$7.27x10^6$	$2.45x10^7$
18	12.48	$C_{16}H_{22}O_8$	341.1236	Coniferyl alcohol-*O*-deoxyhexoside	$3.14x10^6$	$4.64x10^6$	$6.50x10^6$	$4.16x10^6$
19	14.11	$C_9H_{16}O_4$	187.0965	Azelaic acid	$7.95x10^6$	1.17x107	$8.07x10^6$	$1.49x10^7$
20	15.34	$C_{21}H_{20}O_{10}$	431.1118	Kaempferol-*O*-deoxyhexoside	$1.58x10^7$	$1.94x10^6$	$1.93x10^6$	$7.93x10^6$
21	16.29	$C_{18}H_{34}O_5$	329.2329	9, 12, 13 Trihydroxyoctadecenoic acid	$1.14x10^6$	$1.13x10^6$	$1.16x10^6$	$1.78x10^6$
22	22.17	$C_{17}H_{26}O_4$	293.1756	6-gingerol	$4.12x10^6$	$8.80x10^6$	$8.00x10^6$	$7.75x10^6$
23	26.27	$C_{24}H_{52}O_7NP^{\#}$	$540.3293^{\#}$	Lyso-phosphatidilcolina (16:0)	$5.77x10^6$	$1.24x10^6$	$1.15x10^6$	$3.40x10^6$
24	31.70	$C_{16}H_{32}O_3$	271.2273	2-Hydroxyhexadecanoic acid	$4.09x10^6$	$6.28 x10^5$	$4.24x10^5$	$7.24x10^5$
25	33.01	$C_{16}H_{32}O_3$	271.2272	3- Hydroxyhexadecanoic acid	$1.36x10^6$	$5.74 x10^5$	$1.53x10^6$	$2.28x10^6$
26	33.87**	$C_{19}H_{36}O_3$	311.2585	3-Hydroxy-nonadecenoic acid	$3.75x10^5$	$1.05 x10^6$	$4.85x10^5$	$5.49x10^5$
27	34.39**	$C_{19}H_{38}O_3$	313.2740	2-Hydroxynonadecanoic acid	$1.78x10^5$	$3.55 x10^6$	$3.91x10^5$	$3.70x10^5$
28	34.95	$C_{18}H_{36}O_3$	299.2583	3-Hydroxyoctadecanoic acid	$1.40x10^6$	$1.48x10^5$	$1.81x10^5$	$2.92x10^5$
29	35.82**	$C_{19}H_{38}O_3$	313.2740	3-Hydroxynonadecanoic acid	$6.58 x10^4$	$1.58 x10^6$	$4.17x10^5$	$3.02x10^5$
30	36.39**	$C_{20}H_{40}O_3$	327.2894	3-Hydroxyeicosanoic acid	$2.37x10^6$	$1.98x10^6$	$1.91x10^6$	$1.25x10^6$

F: flowers extract; L: leaves extract; S: stem extract; Fr: fruits extract; $^{\#}$ related to $[(M + FA)-H]^-$; related to flowers extract; $**R_t$ related to leaves extract

plants

MDPI

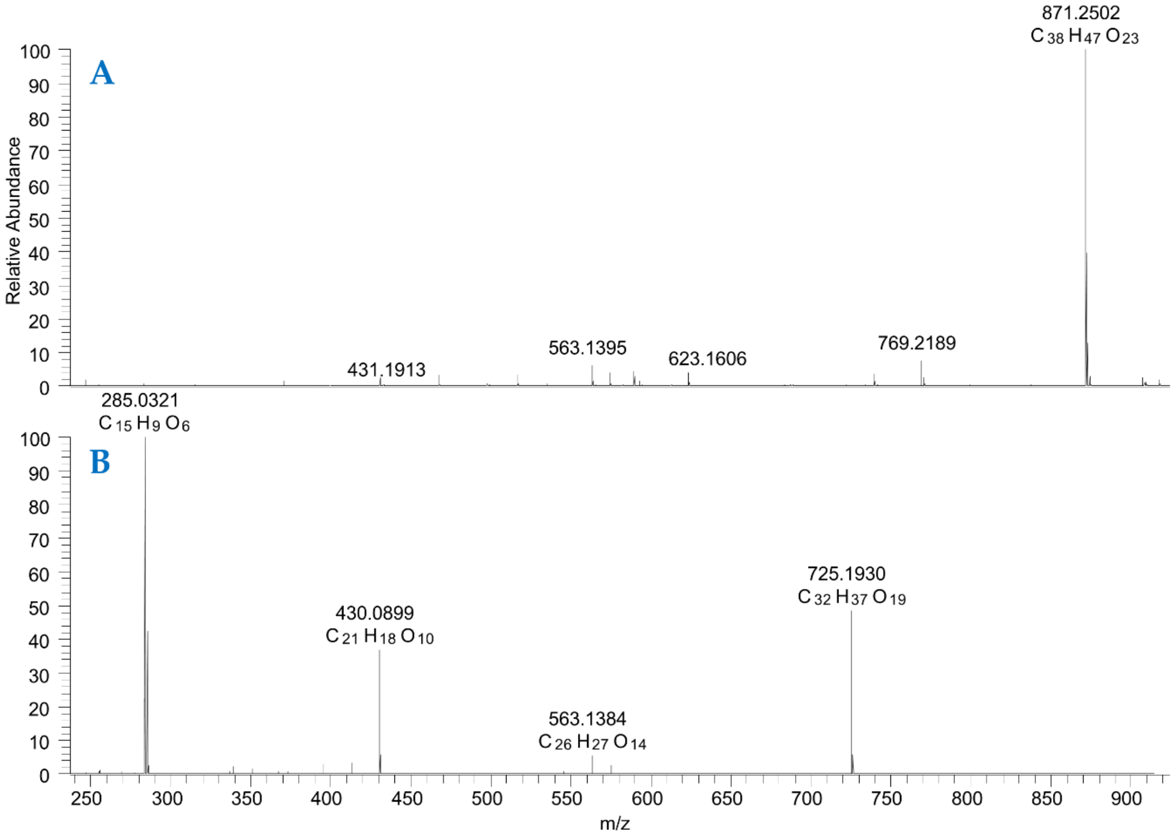

Figure S1. ESI–Q Exactive MS/MS spectrum of compound **12** (A) and its corresponding MS/MS spectrum (B), in negative ion mode.
